# Acoustic transmissive cloaking with adjustable capacity to the incident direction

**DOI:** 10.1038/s41378-022-00448-1

**Published:** 2022-09-28

**Authors:** Meng Lian, Linqiu Duan, Junjie Chen, Jingyuan Jia, Ying Su, Tun Cao

**Affiliations:** School of Optoelectronic Engineering and Instrumentation Science, Dalian University of Technology, 116024 Dalian, China

**Keywords:** Optical materials and structures, Micro-optics

## Abstract

Zero-refractive-index (ZRI) phononic crystals (PhCs), in which acoustic waves can be transmitted without phase variations, have considerable potential for engineering wavefronts and thus are applicable to invisibility cloaking. However, the creation of the transmissive cloaking achieved by ZRI-PhCs is challenging under an oblique incidence, which substantially hinders their practical applications. Here, we experimentally demonstrate acoustic transmissive cloaking with the adjustable capacity to the incident direction. Acoustic transmissive cloaking of arbitrarily shaped obstacles can be obtained through a hybrid acoustic structure consisting of one outer layer of a programmable phase-engineered metasurface (PPEM) and one inner layer of a double zero-refractive-index (DZRI)-PhC. The DZRI-PhC is functionally the same as an equiphase area and can guide acoustic waves around the obstacle, a process known as acoustic tunneling. The PPEM perpendicularly transfers the incident acoustic waves to the DZRI-PhC and allows the emergent waves from the DZRI-PhC to transmit along the original incident direction. The DZRI-PhC is made of an array of iron squares in the air. The reciprocal of the effective bulk modulus and the effective mass density is approximately zero at a frequency of 3015 Hz (0.5187 *v*_0_*/a*) originating from the zeroth-order Fabry–Pérot (FP) resonance that possesses infinite phase velocities. Each meta-atom of the outer metasurface consists of a line channel and four shunted Helmholtz resonators, which have effective masses that are engineered by a mechanics system. The amplitude and phase of the sound waves propagating through each meta-atom can be controlled continuously and dynamically, enabling the metasurface to obtain versatile wavefront manipulation functions. Acoustic cloaking is visually demonstrated by experimentally scanning the acoustic field over the hybrid structure at a frequency of 3000 Hz (0.5160 *v*_0_*/a*). Our work may provide applications with great potential, including underwater ultrasound, airborne sound, acoustic communication, imaging, etc.

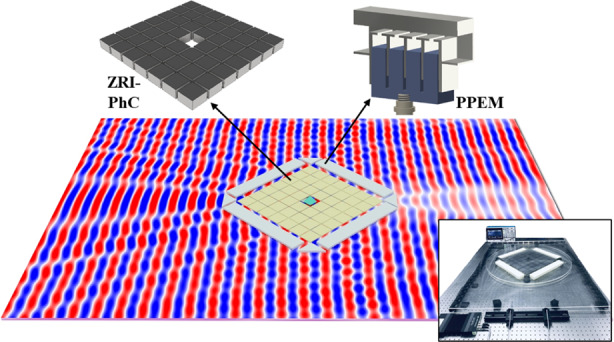

## Introduction

Acoustic cloaking is a phenomenon that causes obstacles to be undetectable to sound waves^1^. Generally, acoustic invisibility is obtained by decreasing the noise produced by a hidden obstacle or employing acoustic absorption materials to prevent echoes from occurring. Acoustic metamaterials (AMMs) have acoustic characteristics that do not exist in nature, i.e., negative bulk modulus^2,3^ and anisotropic mass density^4^, which can engineer sound waves in unique manners with fascinating parameters^5–7^. Recently, acoustic invisibility based upon AMMs has become a fascinating topic because of its ability to avoid impinging acoustic waves from being influenced by the scattering obstacle^8,9^. However, artificial AMMs built by local resonating microstructures possess intrinsic resonance losses^10,11^. On the other hand, phononic crystals also exhibit similar phenomena, such as gradient index focusing, subwavelength imaging, and negative refraction^12–14^. This strategy shuns the local resonance and loss while pushing the operating frequencies to the diffraction region in which a rigorous effective medium is improperly defined^15^. In particular, zero-refractive-index (ZRI) phononic crystals (PhCs) pave another path to obtain transmission-type cloaking^15–17^. A sound wave can transmit with no phase accumulation inside a ZRI-PhC. The acoustic energy could tunnel through a waveguide filled with zero-index material even with a few integrated hidden materials^15,18^. Thus, ZRI-PhCs have a promising ability to shield a scattering obstacle and guide acoustic waves through a bent structure, in which a scatterer positioned inside the hidden area is not audible^18^. However, a waveguide geometry with a rigid wall to assure the vertical incidence to ZRI-PhCs is needed in accordance with Snell’s law^19^. The requirement on the normal input wave significantly forbids the application of ZRI-PhCs.

Recently, optical cloaking has been demonstrated in the microwave region using a ZRI material wrapped by a transparent metasurface^20^. The ZRI material can guide the incident light around the hidden objects and allows the light to dissipate. The outgoing light can be engineered to propagate along the initial incident direction and cause the object to not be detectable. Very recently, an acoustic analogy to the cloaking phenomenon was numerically demonstrated, in which the hybrid cloaking structure was composed of a ZRI metasurface with zero density and a phase-engineer metasurface (PEM) with high transmission^21,22^. Even though they hold great potential for manipulating acoustic waves to achieve cloaking under an oblique incidence, the cloaking phenomenon cannot be maintained by varying the incident angle. This is because when acoustic waves with various incident angles interfere with the subwavelength resonator, the amplitude and phase of the reflected and transmitted waves need to be engineered accordingly. Conventional metasurfaces are composed of meta-atoms with fixed Helmholtz resonators^23,24^ and geometries^15,25^. To achieve the cloaking function under different incident angles using a PEM composed of fixed-geometry unit cells, a different PEM has to be designed and fabricated via time-consuming and costly procedures, which highly limit its practical impacts. Thus, a programmable phase-engineer metasurface (PPEM) is desired since its acoustic response can be engineered to achieve the cloaking phenomenon under various incident angles.

In this work, we both theoretically and experimentally explored, for the first time, a two-dimensional (2D) transmissive acoustic cloaking consisting of ZRI-PhCs and a PPEM. This hybrid structure possesses the adjustable capacity to the incident angle of the acoustic wave. The ZRI-PhCs can achieve acoustic energy tunneling, and the PPEM can dynamically manipulate wavefronts. Our proposed ZRI-PhC can achieve near-zero quantities in both the reciprocal of the effective bulk modulus and the effective mass density. In contrast to prior works^26–29^, our proposed 2D-PhC does not need extreme material parameters, and the near-zero effective medium parameters originate from the zeroth-order Fabry–Pérot (FP) resonance with an infinite phase velocity^18^. In addition, the PPEM is made of a tunable microresonator array, and each unit cell has a line channel integrated with four shunted Helmholtz resonators, in which effective mass/cavity sizes can be controlled using a mechanical system. Based upon this mechanism, the amplitude and phase of transmitted sound waves propagating through each meta-atom can be engineered continuously and dynamically. By wrapping 2D ZRI-PhCs with the PPEM, we experimentally illustrate that the PPEM can allow on-demand modulations of the propagating wave to always be normal to the entrance surface of ZRI-PhCs. This enables ZRI-PhCs to achieve transmissive cloaking under different incident angles. This work may provide progress toward multifunctional and dynamical acoustic devices that require smart and reconfigurable properties.

## Results and discussion

**Fig. 1:**
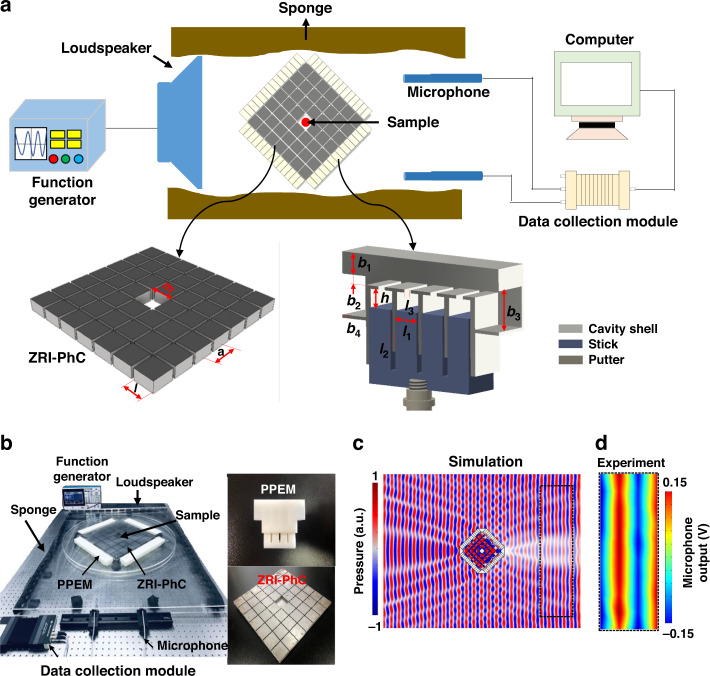
Theoretical and experimental demonstration of acoustic transmissive cloaking using hybrid subwavelength structures. **a** Top panel: schematics of acoustic transmissive cloaking composed of a PPEM and ZRI-PhC. Bottom panel: schematics of the ZRI-PhC and PPEM in section, respectively. **b** Photographs of the experimental setup (left column) and the fabricated structures (right column). **c** Numerical simulation and **d** experimental measurement of a plane wave transmitting through the hybrid cloaking structure under an oblique incident angle of *θ*_*i*_ = 45°. The measured intensity distribution is presented for the area corresponding to the dashed box shown in **c**

A ZRI material can offer versatile intriguing phenomena such as beam-shaping and cloaking^29,30^. Herein, we theoretically and experimentally demonstrate cloaking of an object with arbitrary size and shape using hybrid subwavelength structures. Our proposed structure consists of two layers, as schematically shown in Fig. 1a. The inner gray and beige layers represent the ZRI-PhC and PPEM, respectively. In particular, the ZRI-PhC was developed from a previously reported structure^18^ while operating at lower frequencies. When a sound wave obliquely impinges on the outer PPEM film from the left, the left outer PPEM can guide the transmitted wave vertically to the entrance surface of the PhC. Therefore, the wave can couple into the ZRI-PhCs and squeeze through the ZRI-PhCs with high transmittance because of the tunneling phenomenon. Since the outgoing sound wave is perpendicular to the exit interface of ZRI-PhCs, we also place the PPEM layer on the output (right outer side) of the PhCs to conform the exit sound wavefront to be the same as the incident wave. Consequently, a cloaking functionality can be achieved by compensating for the transmissive phase and correcting the wavefront of the incident wave. The experimental setup exhibits a square hole with a length of *m* = 62 mm that is embedded in the PhCs, exhibiting 7 × 7 steel squares with a pitch of *a* = 59 mm. The steel square has an identical size of *l* = 56 mm. When the frequency of an incident wave is close to the FP resonance of the PhCs, both the effective density and index of the PhCs approach zero, and the effective acoustic velocity is much larger than the velocity in air. Steel is employed in ZRIs because its acoustic impedance is much larger than that of air. The inset presents a reconfigurable unit cell of the PPEM. The unit cell comprises a straight channel integrated with four shunted Helmholtz resonators loaded with acrylic resin sticks controlled by a putter to regulate the cavity size and acoustic response. We first investigate the cloaking effect under an incident angle of *θ*_*i*_ = 45°. The dimensions of the unit cell are *b*_1_ = 7 mm, *b*_2_ = 2.5 mm, *b*_3_ = 13 mm, *b*_4_ = 1 mm, *l*_1_ = 8.2 mm, *l*_2_ = 1.8 mm, and *l*_3_ = 1.5 mm. The pitch of the PPEM is 27.8 mm, which is approximately a quarter wavelength of the acoustic wave with an FP resonance frequency of PhCs (~3000 Hz) to offer a sufficient spatial resolution. Accordingly, our proposed hybrid structure combines the phase-controlling ability of a PPEM and the equal-phase surface feature of ZRI-PhCs to enable sound waves to propagate along the initial direction around the hidden object. The cavity size of a resonator can be easily controlled by moving the acrylic resin stick using a mechanical system. The sponges (brown color) are placed at two sides of the hybrid structure to minimize reflections. A function generator (Tektronix AFG31052) is employed as a sound source with a central frequency of 3000 Hz. In the experiment, an array of loudspeakers is connected to the function generator to produce the incident sound waves. The loudspeaker array is positioned 100 mm away from the left side of the PhCs to send a plane wave source to the hybrid structure. Two microphones (Brüel & Kjær Type 4939-A-011) are located 500 mm away from the right side of the ZRI-PhCs to sense the output sound pressure. The amplitude and phase of acoustic waves are sampled by the data collection module (Brüel & Kjær Type 3052-A-030) with a frequency of 50 MHz. The collected experimental data are subsequently processed using a computer. Figure 1b presents photographs of the experimental setup (left column) and the fabricated structures of the ZRI-PhCs and PPEM (right column). For the initial illustration, we consider a rhombic hybrid cloaking structure. In Fig. 1c, we numerically demonstrate a perfect cloaking phenomenon with small scattering using the numerical package COMSOL Multiphysics based on the finite element method (FEM) (see “Simulation”). A plane wave launched from the left maintains its amplitude and phase continuity after transmitting through the hybrid cloaking structure. A nearly plane wavefront is preserved at the propagated region (circled by a black dashed line). A PPEM layer using the geometrical parameters designed above can convert the 45° incident acoustic wave to be normal to its surface, which ensures that the vertical direction couples to the ZRI-PhC layer. By controlling the local effective sound velocity, various transmission phases ranging from 0 to 2π can be obtained. Accordingly, the local effective mass density can be engineered to match the local impedance at each meta-atom to the acoustic impedance under a 45° incident angle. By using this strategy, high transmittance can be achieved. As a plane wave is imposed on a metasurface layer, the propagated sound wave can be redirected based on Snell’s law:

1 $$\left( {{\mathrm{sin}}\theta _p - {\mathrm{sin}}\theta _i} \right)k_0 = {\mathrm{d}}\varphi /{\mathrm{d}}y$$

where *θ*_*i*_and *θ*_*p*_ represent the incident and transmitted angles, respectively, and *k*_0_ is the wavenumber in air. We discretize the phase gradient along the surface d*φ*/d*y* by fourteen meta-atoms, and the propagated phase variation between neighboring meta-atoms is tuned as π/3. The related propagation angle is approximately *θ*_*p*_ = 0° under *θ*_*i*_ = 45°, which satisfies the design requirement of the rhombic cloaking structure. To achieve sound tunneling, the effective velocity and mass density of the ZRI-PhC are *v*_ZIM_ = 1000 *v*_0_ and *ζ*_ZIM_ = 0.001 *ζ*_0_, respectively. *v*_0_ is the velocity of the sound wave in air, and *ζ*_0_ is the mass density of air. We then explore the performance of a hybrid rhombic cloaking structure composed of the ZRI-PhC and PPEM metasurfaces. Four lines of the PPEM are placed on the four sides of the PhCs to build up the rhombus hybrid cloaking structure. Although both the ZRI-PhCs and PPEM are designed to match the impedance to air, the near-field coupling can damage the cloaking response by placing them closely without an aperture. Thus, a 1 cm air aperture between the metasurface layer and PhC is introduced to improve impedance. In Fig. 1d, we experimentally characterized the acoustic energy and pressure fields of propagating waves through a hybrid rhombic cloaking structure. The geometry of the structure was set to those designed in Fig. 1a. A 3000 Hz acoustic wave came from the left and impinged on the PhC containing the object. The details of the experimental measurement can be found in the “Experimental Section”. The wavefronts in the experimental pressure field (Fig. 1d) are in excellent agreement with those in the numerical simulation. Despite the imperfection from the plane wave source and boundary reflections, it is evident that the wavefront maintains its initial pattern after it propagates through the ZRI-PC as if it is not there. We numerically investigated the influence of the position of the air square on the intensity distributions of the sound pressure at *f* = 0.5190 *v*_0_/a. As shown in Supplementary Fig. S1, the patterns of the pressure field of the PhC were slightly distorted by varying the position of the air square. However, this can be further improved by constructing PhCs with more layers of steel squares, such as 9 × 9 steel squares, as shown in Supplementary Fig. S2.

**Fig. 2:**
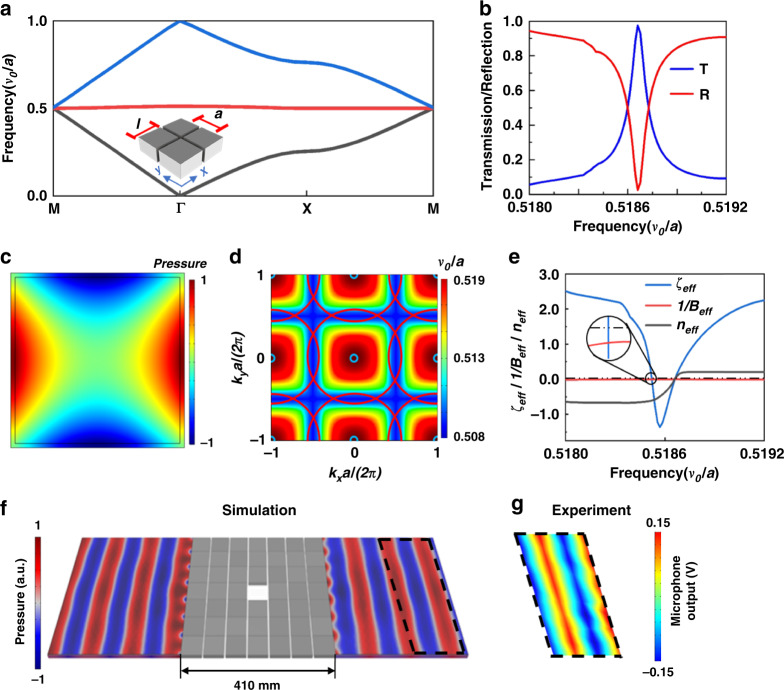
A 2D ZRI-PhC composed of a steel squares array in air. **a** Numerically calculated band structure of the ZRI-PhC. **b** Experimentally measured transmittance and reflectance. **c** The pressure-field pattern of a single resonator at *f* = 0.5187 *v*_0_/*a*. **d** The iso-frequency contour of the flat band. At a working frequency of *f* = 0.5187 *v*_0_/*a*, the iso-frequency contour is a small cylinder (circled by a cyan line). The red circle represents the iso-frequency contour of air. **e** The spectra of effective medium parameters (1/*B*_*eff*_, *ζ*_*eff*_, and *n*_*eff*_) of the PhC. The *ζ*_*eff*_ experiences a sharp change around *f* = 0.5186 *v*_0_/*a*. **f**, **g** The (**f**) numerically simulated and (**g**) experimentally measured intensity distributions of the sound pressure at *f* = 0.5190 *v*_0_/*a* and *f* = 0.5160 *v*_0_/*a*, respectively. The measured intensity distribution is presented for the area corresponding to the dashed box shown in **g**

Herein, the 2D ZRI-PhC is composed of a steel squares array in air. A schematic of the PhC with four atoms is presented in the inset of Fig. 2a. The dimension of the steel square is infinite along the *z* axis. Along the *x* and *y* axes, the steel square has an identical size of *l* = 0.95*a*, where *a* is the lattice constant of the PhCs. The mass densities of air and steel are ζ_0_ = 1.25 kg/m^3^ and ζ = 7870 kg/m^3^, respectively. Their sound velocities are *v*_0_ = 343 m/s and *v* = 5960 m/s, respectively. The dispersion relation of the ZRI-PhC is simulated and presented together with the measured transmission (blue line) and reflection (red line) of the ZRI-PhCs. In Fig. 2a, we calculate the band structure of ZRI-PhCs by using the commercial software COMSOL Multiphysics. Thus, a flat mode band occurs around the frequency of 0.5187 *v*_0_*/a* over the whole Brillouin zone, which does not intersect with the other mode bands. Both distinct peaks and valleys were experimentally observed in the transmission and reflection spectra, respectively, as shown in Fig. 2b. This indicates the excitation of the FP cavity resonance in the PhCs. It was found that the flat mode at 0.5187 *v*_0_*/a* for ZRI-PhCs perfectly matched the measured peak transmission/dip reflection. The pressure-field distribution of the eigenmode on the flat band at the Γ point is plotted in Fig. 2c. Because of the large impedance mismatch between air and steel, the sound wavefield is localized efficiently in the air apertures. To further explore the fundamental physics, the iso-frequency contour of the straight mode band at 0.5187 *v*_0_*/a* is analyzed. As presented in Fig. 2d, the iso-frequency contour appears to be circular around the Γ point, while it becomes more rectangular as the frequency decreases. Particularly, at a working frequency of 0.5187 *v*_0_/*a*, the iso-frequency curve is a small cylinder (circled by the cyan line), indicating that the PhC is an effective isotropic medium. In the vicinity of the center of the Brillouin zone, the ZRI-PhC can be featured by an effective medium theory (EMT) since the wave vector $\vec k$is close to zero^31^. Namely, the PhC can be expressed by the effective bulk modulus *B*_*eff*_ and effective mass density *ζ*_*eff*_^32^, which are extracted from the numerically simulated complex transmission and reflection coefficients^32^. In Fig. 2e, we present the spectra of 1/*B*_*eff*_ (red line), *ζ*_*eff*_ (blue line), and effective refractive index *n*_*eff*_ (green line) in the spectral region from 0.5180 *v*_0_*/a* to 0.5192 *v*_0_*/a*. As seen in the inset circle, the 1/*B*_*eff*_ is negative and approaches zero over the whole spectral region. In contrast, *ζ*_*eff*_ drastically changes from positive to negative after it gradually increases and becomes positive. Both 1/*B*_*eff*_ and *ζ*_*eff*_ are negative in the spectral region from 0.5180 *v*_0_*/a* to 0.5186 *v*_0_*/a*, producing a negative band. At *f* = 0.5187 *v*_0_*/a*, *ζ*_*eff*_ and *n*_*eff*_ simultaneously reach zero with nearly unity transmission, which indicates that the PhC exhibits a zero-refractive index property whose impedance matches that of air. In Fig. 2f, g, we both numerically and experimentally show the patterns of the pressure field of the PhC with an area of 410 × 410 mm^2^, respectively. In the simulation, the working frequency is 0.5190 *v*_0_/*a* (*f* = 3017 Hz), which is slightly larger than 0.5187 *v*_0_/*a* because of the small variation in impedance when the obstacle is placed inside the PhC. In the measurement, a sound wave with a frequency of 0.5160 *v*_0_*/a* (*f* = 3000 Hz) is launched from the left and imposed on the PhC, encompassing the obstacle. Regardless of the defect originating from the boundary reflections and plane wave source, the wavefront can maintain its original pattern as transmitting through the PhC as if the steel squares array was absent. The measurement is in excellent agreement with the simulation. This shows that the PhC contributes the most to hide the object. There is a small difference in frequency between the simulation and experiment. Such a discrepancy was mainly caused by experimental uncertainties, fabrication errors, and the thermoviscous effect that are not considered in the numerical model^33,34^. In Supplementary Fig. S3, we have both simulated and measured a plane wave transmitting through the obstacle with and without a cloaking structure, respectively. As shown, a nearly plane wavefront was preserved at the propagated region using a hybrid cloaking structure (Supplementary Fig. S3a), while the wavefront propagating through the obstacle was seriously distorted without cloaking (Supplementary Fig. S3b).

**Fig. 3:**
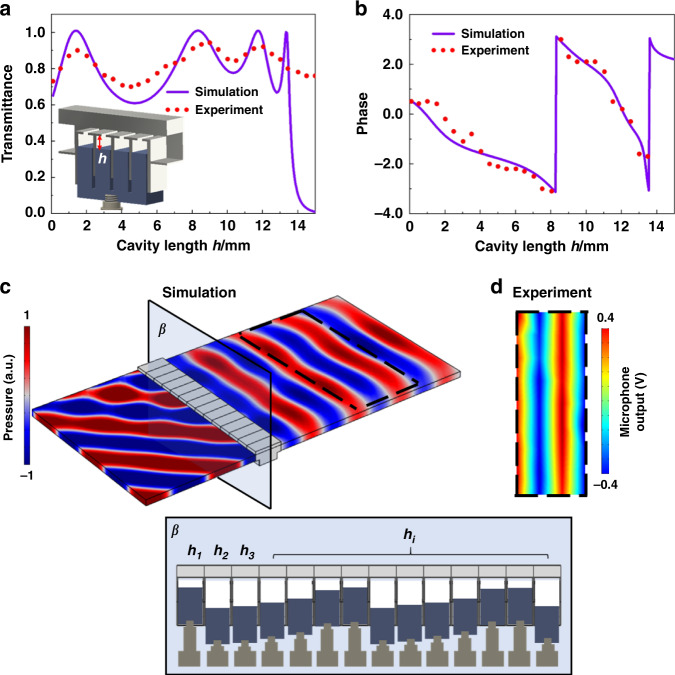
Real-time control of a reconfigurable unit cell. **a** Amplitude and **b** phase of propagated sound waves with respect to cavity length *h*. **c** Simulated and **d** measured pressure-field distributions for the PPEM, which bends an acoustic wave by 45° at *f* = 0.5190 *v*_0_/*a* and *f* = 0.5160 *v*_0_/*a*, respectively. The measured pressure-field distribution is presented for the area circled by the dashed line in **c**

To design a PPEM with a subwavelength thickness and the capability to cause oblique incidences to be vertical to the surface of the ZRI-PhC, an acoustic resonator composed of four shunted Helmholtz resonators and a straight pipe is employed, as shown in Fig. 1a. In Fig. 3, we first fabricate a single-unit cell of a PPEM and experimentally examine its acoustic feature during real-time controlling. The cavity length (*h*) of the shunted Helmholtz resonator is modulated by gradually moving the acrylic resin sticks inside the cavity. A cross-sectional view of the unit cell is presented in the inset of Fig. 3a. A phase change over a full 2π range can be obtained by varying *h*. The loudspeaker launches the plane wave to the unit cell, and the microphones detect the propagating sound waves during the alternation of *h*. A Fourier transform is applied to the acquired signals to extract the phase and amplitude of the propagated waves. In Fig. 3a, we both experimentally (red dots) and numerically (purple line) show that the unit cell can maintain a high transmission amplitude as *h* increases from 0.1 to 15 mm. In Fig. 3b, we explore the *h*-dependent transmission phase for each meta-atom. As observed, a complete 2π phase modulation can be obtained by gradually changing *h* from 0.1 to 13 mm. The measured transmission phases match well with the numerically simulated phases. The measurements show that our proposed mechanical-actuated reconfigurable resonator can dynamically and continuously obtain a programable phase. In the simulation, the fabrication tolerance, experimental uncertainties, and thermoviscous effect^33,34^ are ignored. This induces the difference between the measurement and simulations. To achieve a rhombic hybrid cloaking structure, the PPEM is required to convert the incident wave with angles of *θ*_*i*_ = 45° to be normal to the surface of the ZRI-PhCs. A phase gradient d*φ/*d*y* = −1.414π/*λ*_0_ is taken to bend the sound wave by 45°, where *λ*_0_ represents the wavelength of sound waves in air. We consider a discretization of fourteen sets of meta-atoms whose cavity lengths (*h*) are shown in Table 1. Figure 3c, d experimentally and numerically illustrates the pressure-field distributions when the sound wave propagates through the PPEM at *f* = 0.5190 *v*_0_/*a* and *f* = 0.5160 *v*_0_/*a*, respectively. It is found that an input 420 mm broad plane wave can be bent by 45° by the PPEM. The wavefront of the propagated wave is almost parallel to the surface of the PPEM.

**Table 1:** The parameters *h* (mm) of fourteen sets of meta-atoms under different *θ*_*i*_ values

*θ*_*i*_	*h*_1_	*h*_2_	*h*_3_	*h*_4_	*h*_5_	*h*_6_	*h*_7_	*h*_8_	*h*_9_	*h*_10_	*h*_11_	*h*_12_	*h*_13_	*h*_14_
0°	10	10	10	10	10	10	10	10	10	10	10	10	10	10
30°	7.5	3.1	1.9	13.7	13.7	12.5	11.8	9.8	7.9	3.1	1.9	13.9	12.4	11.8
45°	12.2	8.9	3.3	2.5	9.2	7.1	11.4	8.9	4.2	2.5	7.5	12.7	11.5	9.4
60°	13.9	13.3	9.8	7.1	1.9	13.7	11.9	9.2	6.8	1.4	13.4	11.2	8.6	3.7

**Fig. 4:**
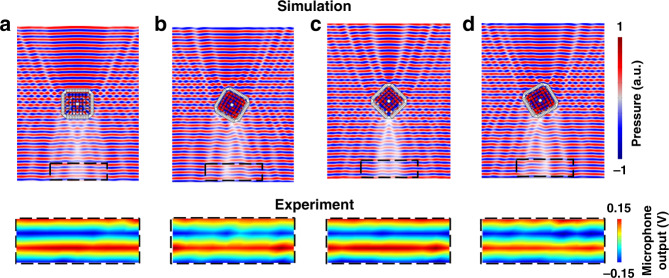
3D-FEM simulation (top panel) and measurement (bottom panel) of sound-pressure field distribution at *f* = 0.5190 *v0/a* and *f* = 0.5160 *v0/a*, respectively. Sound wave transmitting through the hybrid subwavelength structure composed of the PPEM and ZRI-PhC at **a** *θ*_*i*_ = 0°, **b** *θ*_*i*_ = 30°, **c** *θ*_*i*_ = 45°, and **d** *θ*_*i*_ = 60°. The measured pressure-field distribution is presented for the area corresponding to the dashed box shown in the top panel

Ideally, the cloaking function should not depend on the incident angle of the acoustic wave. As discussed above, our proposed reconfigurable unit cell of the PPEM has the advantages of maintaining a high transmission amplitude and engineering the transmission phase from −π to +π by continuously altering the cavity sizes of Helmholtz resonators using a mechanical system. Such an advantage lays the foundation for cloaking under different incident angles. By attaching the PPEM shell to the outer surface of the ZRI-PhC, cloaking under different incident angles can be simply achieved by reconfiguring the unit cell. In Fig. 4, we demonstrate cloaking phenomena under various incident angles using the hybrid subwavelength structure composed of the PPEM and ZRI-PhCs. The top and bottom panels show the simulated and measured pressure-field distributions. The different incident angles of *θ*_*i*_ = 0°, 30°, 45°, and 60° are obtained by rotating the structure clockwise. The acoustic waves mainly transmit along the initial incident direction under different *θ*_*i*_ values, and the scattered waves in the other directions can be efficiently restrained. Thus, the cloaking effect can be achieved for arbitrary incident angles by varying the cavity size of Helmholtz resonators. The cavity length *h* of fourteen sets of meta-atoms under different incident angles *θ*_*i*_ are presented in Table 1, accordingly satisfying the phase gradient requirement for the cloaking functionality. Namely, for various *θ*_*i*_ values, the cloaking shell can ideally correct the phase advance while modulating the amplitude to agree with the ideal pressure fields. The experimental measurement of the sound-pressure field matches the simulated data well. Therefore, this hybrid structure can cloak arbitrarily shaped objects over large incident angles.

## Conclusion

In summary, we experimentally achieved a 2D cloak that reduces the visibility of hidden obstacles for airborne acoustic waves with different incident angles. The cloak was obtained by wrapping a PPEM shell around ZRI-PhCs. The ZRI-PhC can achieve the tunneling effect, and the PPEM can dynamically redirect the wavefront. The inner ZRI-PhC is constructed by closely packing the same steel squares and possesses the dispersion feature of a flat mode band across the Brillouin zone around *f* = 0.5187*v*_0_/*a*. This flat band originates from the zeroth-order FP resonance, providing an infinite phase velocity in air channels. Thus, the phase does not change when the acoustic waves propagate through the PhC, indicating an effective zero index. The PPEM is composed of reconfigurable subwavelength resonators for programming sound waves and manipulating wavefronts. By continuously varying the cavity sizes of the Helmholtz resonators with a robust mechanical system, the subwavelength meta-atom can dynamically control the amplitude and phase of the transmission sound waves. This enables the PPEM to convert the incident wave with any incident angle (*θ*_*i*_) to be vertical to the surface of the ZRI-PhC. By experimentally scanning the field distributions of sound pressures at *f* = 0.5160*v*_0_/*a* and energy controlled with the PPEM, we visually illustrate that the incident waves with various *θ*_*i*_ values can go around the objects and transmit along the initial incident direction. In contrast, scattering waves in the other directions are repressed. This work may provide versatile approaches to achieve integrated sound cloaking relying upon hybrid periodic structures with multiple functions.

## Methods

### Fabrication

The ZRI-PhC is constructed by assembling 7 × 7 steel squares with a lattice constant of *a* = 59 mm, in which the steel squares array is fabricated by using computerized numerical control tools. The PhC possesses an air block with a size of 62 × 62 mm in which the object can be placed. The PPEM resonators are fabricated by 3D polyjet printing with resins that have a much larger acoustic impedance than air. The material of the PPEM is resin. The PPEM is composed of fabricated resonators periodically distributed with a pitch of 27.8 mm. The cavity length of the Helmholtz resonators in the metasurface can be modulated by moving the resin stick into/out of the cavity resonators.

### Measurements

The sound wavefield is acquired in an area of 0.8 m × 0.2 m with two motorized arms that scan in the *x*- and *y* directions employed for the sonic scanning measurement. We controlled the scanning system using the FlexSCAN-C system from Sonix. The experimental setup is illustrated in Fig. 1. An incident plane wave was produced by an array of 10 loudspeakers that were placed 100 mm away from the hybrid cloaking structure. A function generator (Tektronix AFG31052) produced sinusoidal waves to the array of the speakers. Two free-field microphones with a diameter of 6.35 mm (B&K Type 4939-A-011), which were placed 500 mm away from the output side of the cloaking structure, were used as receivers. We digitized the received data using a FlexSCAN-C system with a sampling rate of 50 MHz and a resolution of 16 bits. The amplitudes of the received signal were collected at all scanning points at identical time points after receiving ten cycles. We acquired 2D wavefields of the propagated sound waves by point-by-point scanning in an area of 0.8 m × 0.2 m with a step of 0.01 m.

### Simulation

The numerical simulations of a unit cell were performed using the pressure acoustics module from the commercial software COMSOL Multiphysics based on the finite element method. We defined the background medium as air that has a density of 1.2 kg/m^3^ and a speed of sound of 343 m/s. The boundary conditions of the unit cell are rigid for intensive sound scattering owing to the mismatched impedance. To extract the transmission coefficients of the meta-atom, we launched an incident plane wave to one side of the meta-atom and placed perfectly matched layers on the other side to reduce reflections. We employed a probe in the outlet of the straight channel to extract the transmission amplitude at the various cavity sizes. To simplify the simulation, the loss effect is neglected.

## Supplementary information

supporting information
