# Supplementary material for: Acoustic transmissive cloaking with adjustable capacity to the incident direction
Source: Microsyst Nanoeng. 2022 Sep 28;8:108. doi: 10.1038/s41378-022-00448-1 (PMC9515090; doi:10.1038/s41378-022-00448-1)
Supplement: Supplementary file 1 — supporting information [file 41378_2022_448_MOESM1_ESM.docx]

**Supporting Information**

Acoustic transmissive cloaking with adjustable capacity to incident direction

Meng Lian^a,b^, Linqiu Duan^a^, Chen Junjie^a^, Jingyuan Jia^a^, Ying Su^a^, and Tun Cao*^a,b^

^a^ School of Optoelectronic Engineering and Instrumentation Science, Dalian University of Technology, Dalian 116024, China

^b^ These authors equally contributed to the work

*Corresponding author: [caotun1806@dlut.edu.cn](mailto:caotun1806@dlut.edu.cn)

**Figure** S1. The numerically simulated intensity distributions of the sound pressure at *f* = 0.5190 *v*_0_/a as placing the air square in the (a) top, (b) left, (c) bottom, (d) right of the center of the ZRI-PhC composed of 7×7 steel squares.

**Figure** S2. The numerically simulated intensity distributions of the sound pressure at *f* = 0.5190 *v*_0_/a as placing the air square in the (a) top, (b)left, (c) bottom, (d) right of the center of the ZRI-PhC composed of 9×9 steel squares.

**Figure** S3. The numerical simulation (top panel) and experimental measurement (bottom panel) of a plane wave transmitting through the (a) hybrid cloaking structure and (b) obstacle under an oblique incident angle of *θ_i_* = 45°. The measured intensity distribution is presented for the area corresponding to the dashed box shown in top panels.
